# Comparison of Cost-Effectiveness between Inpatient and Home-Based Post-Acute Care Models for Stroke Rehabilitation in Taiwan

**DOI:** 10.3390/ijerph18084129

**Published:** 2021-04-14

**Authors:** Yu-Ju Tung, Wen-Chih Lin, Lin-Fu Lee, Hong-Min Lin, Chung-Han Ho, Willy Chou

**Affiliations:** 1 Department of Physical Medicine and Rehabilitation, Chi Mei Medical Center, Tainan 710, Taiwan; roofyyuju@gmail.com; 2 Department of Physical Medicine and Rehabilitation, Chi Mei Medical Center, Chiali Branch, Tainan 722, Taiwan; mosmalin@gmail.com (W.-C.L.); 911044@mail.chimei.org.tw (L.-F.L.); 3 Institute of Medical Education, Chi Mei Medical Center, Tainan 710, Taiwan; geography6000@gmail.com; 4 Department of Medical Research, Chi Mei Medical Center, Tainan 710, Taiwan; ho.c.hank@gmail.com

**Keywords:** stroke, post-acute care, cost-effectiveness, rehabilitation, functional recovery

## Abstract

Stroke rehabilitation focuses on alleviating post-stroke disability. Post-acute care (PAC) offers an intensive rehabilitative program as transitional care following acute stroke. A novel home-based PAC program has been initiated in Taiwan since 2019. Our study aimed to compare the current inpatient PAC model with a novel home-based PAC model in cost-effectiveness and functional recovery for stroke patients in Taiwan. One hundred ninety-seven stroke patients eligible for the PAC program were divided into two different health interventional groups. One received rehabilitation during hospitalization, and the other received rehabilitation by therapists at home. To evaluate the health economics, we assessed the total medical expenditure on rehabilitation using the health system of Taiwan national health insurance and performed cost-effectiveness analyses using improvements of daily activity in stroke patients based on the Barthel index (BI). Total rehabilitative duration and functional recovery were also documented. The total rehabilitative cost was cheaper in the home-based PAC group (*p* < 0.001), and the cost-effectiveness is USD 152.474 ± USD 164.661 in the inpatient group, and USD 48.184 ± USD 35.018 in the home group (*p* < 0.001). Lesser rehabilitative hours per 1-point increase of BI score was noted in the home-PAC group with similar improvements in daily activities, life quality and nutrition in both groups. Home-based PAC is more cost-effective than inpatient PAC for stroke rehabilitation.

## 1. Introduction

Stroke is a common cause of disability and morbidity associated with increased economic burden [1]. Among patients with stroke, 15–30% face permanent disability, and 20% require long-term care [2]. Complications of stroke include limb weakness, low physical fitness, sensory defects, dysphagia, aphasia, poor coordination, cognitive impairment, anxiety, and depression [3,4,5,6]. The American Heart Association/American Stroke Association recommends rehabilitation as a primary mechanism for functional recovery and independence in patients with acute stroke [7]. Because caring for patients with stroke-related disabilities exerts considerable physical, psychological, and economic burden on healthcare systems, caregivers, and societies in general [8], stroke rehabilitation is initiated in the acute stage. Post-acute care (PAC) is designed to maximize functional recovery.

PAC is a phase marking the transition of patients from being in the hospital to returning home, and its goal is to help patients achieve the highest possible functional level [7]. Post-acute stroke rehabilitation offers intensive therapeutic courses after acute medical treatment. This type of PAC can be offered by long-term care hospitals, inpatient rehabilitation facilities, skilled nursing facilities, and home health agencies; in the United States, 62.6–74.5% of patients with stroke are enrolled in such programs [9]. In 2014, Taiwan’s national health insurance program introduced inpatient PAC programs for eligible patients with stroke. These involve referral to a local or district hospital from an acute medical unit for physical, occupational, and speech therapy. Taiwan’s inpatient PAC programs have resulted in significant improvements in patients’ activities of daily living, cardiopulmonary capacity, cognition, nutrition, oral function, balance, speed of walking, language abilities, mobility, and sensorimotor function [2,10,11]. These programs also increase the success rates removing urinary catheters and nasogastric tubes [12], reducing the occurrence of emergency department visits, and reducing the risk of stroke-related admission within 90 days [2]. They are a cost-effective strategy [11,13,14] for subacute care for patients with stroke.

As the stroke mortality rate decreases in Taiwan [15], the need for stroke subacute care increases. In 2019, a novel rehabilitative model—home-based PAC—was initiated in Taiwan. This model transformed hospital-oriented rehabilitation into rehabilitation set in a domestic, familiar environment, in which patients with stroke receive task-oriented rehabilitative training in their homes from therapists delivering remedial courses. Generally, home-based rehabilitation in the United States is conducted by home healthcare agencies and outpatient offices or clinics, and it fulfills both the medical needs of patients and their social desire to return home [16]. Home-based programs also lead to greater independence, performance in daily living activities [17,18,19] and satisfaction than do institutional-based rehabilitation programs [17,18,19,20,21,22]. A previous study revealed similar patients’ perceived performance, satisfaction and difficulty in daily function in home-based and conventional rehabilitation in hospital after occupational therapy [23]. Home-based PAC programs can facilitate patient- and caregiver-centered rehabilitation and promote the transfer of skills learned from training programs into daily practice and attracts patients with its familiar environment (which may lead to better sleep and mood), decrease transportation needs/time between home and outpatient rehabilitative center, easier for the family taking care of patients and increasing of healthcare accessibility. On the other hand, previous studies showed rehabilitation cost in day hospital service was 25% more than domiciliary service for poststroke patients discharged from geriatric ward and 2.6 times more in domiciliary service as compared with outpatient departments for patients discharged from a stroke unit in western countries [24]. Home-based PAC programs have been inaugurated in terms of better functional recovery and lesser medical cost in Taiwan, and our hospital embarked on most of the home-based PAC cases in Taiwan. To date, limited data evaluated these two different health intervention ever since it launched in 2019; therefore, in this study, we delivered rehabilitative therapy for stroke patients by the same executive faculty and evaluated the cost-effectiveness and functional recovery of the novel home-based PAC model compared with current inpatient PAC settings.

## 2. Materials and Methods

### 2.1. Study Design and Participant Recruitment

**Figure 1:**
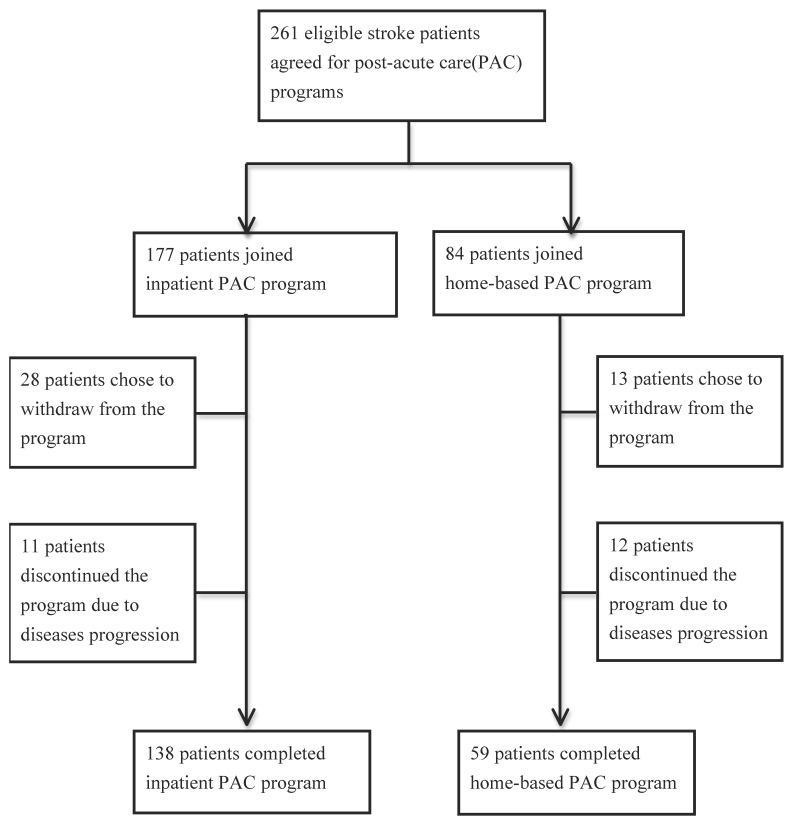
Enrollment in the study.

This retrospective study included patients with stroke referred to Chi Mei Medical Center for post-acute stroke rehabilitation; their eligibility for the program was based on the following inclusion criteria: (1) acute onset of stroke (≤1 month); (2) relatively stable medical condition, with no neurological or hemodynamic deterioration in the past 3 days and no progression of diseases or complications; (3) modified Rankin scale (mRS) score of 3 or 4; and (4) rehabilitative potential, including high motivation, fair consciousness, and adequate physical fitness (being able to sit for 1 h). We excluded patients, who refused to participate in the PAC program, chose to withdraw from the program or discontinued the program due to disease progression (Figure 1). The patients were provided with inpatient PAC or home-based PAC based on their and their families’ willingness and expectations. Finally, 138 patients receiving inpatient PAC and 59 patients receiving home-based PAC, who completed a comprehensive PAC program from June 2015 to December 2020 in our hospital, were recruited. Patients’ clinicodemographic characteristics, health condition, and functional improvement level before and after the PAC program were recorded. This study was approved by the institutional review board of Chi Mei Medical Center.

### 2.2. Intervention

In the inpatient PAC model, patients with stroke were hospitalized for intensive rehabilitation. They received physical, occupational, and speech therapy for 3 h per day on weekdays according to their needs. Physiatrists, therapists, nurses, psychologists, social workers, nutritionists, and medical technicians formed a multidisciplinary team for these patients. In the home-based PAC model, the patients stayed at home, and therapists delivered 50-min rehabilitative sessions six times per week. This model emphasized the use of domestic tools for task-oriented training, merging training programs with daily practical, real-life circumstances, inviting family participation, reassuring caregivers regarding their performance, creating a familiar environment, offering feasible and easy-to-practice activities, and encouraging community engagements. The weekly rehabilitative hours were 15 and 5 h in the inpatient and home PAC model, respectively. Patients’ participation in the program ceased when no functional improvement was noted in two consecutive evaluations, and the PAC team determined that the patient had no potential for functional recovery.

### 2.3. Functional Outcomes

Scores on the mRS indicate stroke severity and the general condition of a patient with stroke, with scores ranging from 0 (no symptoms) to 6 (death). An mRS score of 3 implies moderate disability (i.e., the patient requires some help but can walk unassisted), and an mRS score of 4 implies moderate to severe disability (i.e., the patient is unable to walk or attend to their own bodily needs without assistance) [25]. The Barthel index (BI) reflects a patient’s mobility and self-care ability and includes 10 aspects of activities: feeding, transfer, ambulation, stair climbing, dressing oneself, bowel and bladder control, self-hygiene, toilet use, and showering. The maximum number of points that can be obtained is 5 in the self-hygiene and showering domains; 10 in the feeding, toilet use, dressing, bowel function, bladder control, and stairs climbing domains; and 15 in the ambulation and transfer domains. Thus, the BI score can range from 0 (least independent) to 100 (most independent) [26]. Functional evaluating tools are restricted in the domiciliary environment, and BI, though it may not be flawless, is chosen because it could still be an illustrative and handy assessment for presenting patients’ general function. The Lawton–Brody instrumental activities of daily life scale (IADL) describes a patient’s ability to perform advanced everyday activities, containing eight items on making phone calls, shopping, preparing food, housekeeping, doing laundry, taking pills, and managing finances [27] with numerical scores are assigned from worst to best. The Taiwanese version of the EuroQoL EQ-5D (ED5Q) accurately indicates health-related quality of life based on a patient’s perceptions of five aspects—ambulation, self-care, the conduct of common activities, level of pain and discomfort, and level of anxiety and depression. Each aspect is given a score from 1 to 3 from best to worst quality of life [28,29,30]. The mini nutritional assessment (MNA), mainly used for older adults, has four parts: anthropometric, general, self and dietary evaluations. MNA score ≤ 23.5 indicates a patient at risk of malnutrition [31]. In our study, BI, IADL, ED5Q and MNA were evaluated at the beginning and the end of the PAC program by the same trained therapist in each patient. Improvement in BI, IADL, ED5Q and MNA scores were defined as the difference between the score after PAC completion and that before PAC initiation.

### 2.4. Costs and Health Economics

The economic evaluation was made from national health system perspectives offered by the Taiwan national health insurance. The total cost of rehabilitation of each patient was extracted from declared medical expenses, which were calculated using a payment standard based on Taiwan national health insurance rules; the costs were NTD 3587 (USD 119.57) per rehabilitative day per patient in the inpatient and NTD 1455 (USD 48.50) per session per patient in the home-based model. Cost-effectiveness was calculated as the total cost divided by the improvement in BI, IADL, ED5Q and MNA scores. The intensity of the program was represented by the total rehabilitative hours during the entire PAC program, and the intensity divided improvements in BI score were calculated to evaluate time requirement per 1-point increase of BI.

### 2.5. Statistical Analyses

Continuous variables from functional scales are presented as the mean ± standard deviation. An independent-samples t-test or Wilcoxon rank-sum test was performed for intergroup comparison of continuous variables based on the results of the normalization test. Pearson’s chi-squared test was used for categorical variables. NTD values were converted into USD based on the mean exchange rate over 2019–2020 (30 NTD = 1 USD). Statistical significance was set as *p* < 0.05. All statistical analyses were conducted using SPSS version 21.0 (IBM Corp, Armonk, NY, USA).

## 3. Results

We included 138 and 59 patients with stroke in the inpatient and home PAC models, respectively. Table 1 presents their baseline clinicodemographic characteristics. The inpatient and home groups had similar sex compositions (55.10% vs. 54.24% of patients were male, *p* = 0.914), types of stroke (82.61% vs. 74.58% of patients had ischemic stroke, *p* = 0.195), general condition and severity of stroke (71.74% vs. 61.02% of patients had an mRS score of 4, *p* = 0.238), intensive care unit (ICU) utilization (38.41% vs. 33.90% patients transferred to ICU, *p* = 0.525) and duration from stroke onset to PAC initiation (14.230 ± 7.301 vs. 14.070 ± 6.635 days, *p* = 0.882). There were no significant differences in comorbidities (Charlson comorbidity index and comorbid cardiovascular conditions) or healthcare utilization in the year before the stroke event. Upon discharged from acute ward to PAC programs, proportions of nasogastric tube and Foley catheter used were similar in both groups (13.04% vs. 5.08%, *p* = 0.097 and 7.25% vs. 6.78%, *p* = 0.907), as well as similar ADL function (*p* = 0.157). The median age of stroke acquisition and duration of the PAC program were significantly higher in the home group than in the inpatient group (67.5 years vs. 68 years, *p* = 0.046 and 35.65 vs. 27.00 days, *p* < 0.001). As for the patients who did not complete the PAC program (Figure 1), 28 patients withdrew inpatient PAC program under self-willingness with a mean age of 70.43 years old and 11 patients averagely aged 68.42 who discontinued the program due to disease progression. Meanwhile, 13 patients withdrew home-based PAC program by choice; 12 patients discontinued the program due to disease progression with a mean age of 76.08 versus 71.58 years, respectively.

**Figure 2:**
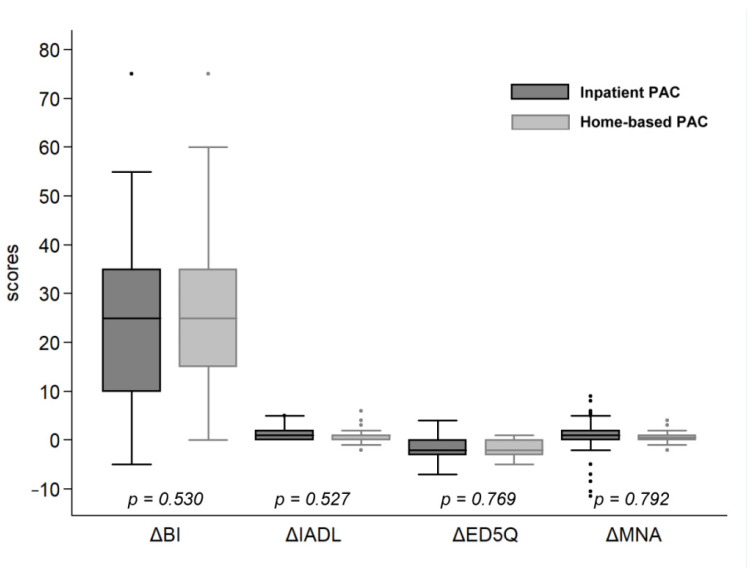
Functional improvements of daily activities, life quality and nutrition between inpatient and home-based PAC groups. **·**: outlier for inpatient PAC; **:**: outlier for home-based PAC.

Table 2 presents the improvements in total BI score and its subdomains. Improvements in BI score were 24.239 ± 16.610 in the inpatient PAC group and 25.667 ± 15.140 in the home PAC group (*p* = 0.530). The improvements in the following domains were similar in both groups: feeding (*p* = 0.265), transfer (*p* = 0.717), ambulation (*p* = 0.843), stairs climbing (*p* = 0.953), bladder control (*p* = 0.659), bowel control (*p* = 0.157), and toilet use (*p* = 0.133). However, the home PAC group exhibited significantly greater improvements in the dressing oneself (*p* = 0.003), self-hygiene (*p* = 0.013), and showering (*p* = 0.001) domains than those in the inpatient PAC group. Age and improvements in BI were not significant different in both groups (Table 3). The improvements in IADL (*p* = 0.527), ED5Q (*p* = 0.769) and MNA (*p* = 0.792) were similar in two PAC groups (Figure 2).

The total rehabilitative cost in Table 4 was significantly different between the inpatient and home groups (*p* < 0.001). The cost-effectiveness versus BI, IADL, ED5Q and MNA were also significantly different in both groups (*p* < 0.001, *p* < 0.001, *p* < 0.001, *p* = 0.001). The total number of rehabilitative hours was significantly higher in the inpatient group (57.86 ± 24.52 vs. 25.39 ± 7.29, *p* < 0.001) despite a shorter length of stay in PAC. When the total numbers of rehabilitative hours were divided by the BI score improvement, a significant difference was noted between the two groups (3.23 ± 3.38 h for the inpatient PAC model and 1.23 ± 0.90 h for the home-based PAC model, *p* < 0.001, Table 4).

## 4. Discussion

Our study compared inpatient and home-based models of subacute stroke rehabilitation and found similar functional recovery in both models, with the home-based PAC model being more cost-effective. Most studies have found this comparison to be challenging because certain institutions or health agencies provide only one rehabilitative model. The results in our study are robust because we compared the functional improvement in two rehabilitative models executed by the same rehabilitative faculty. Our data indicate that improvements in total BI score and in scores for most of the subdomains—feeding, transfer, ambulation, stairs climbing, bladder control, bowel control, and toilet use—were not significantly different between the home-based PAC and inpatient PAC models. A 20-point change in BI score was used as an indicator of significant functional improvement [32], and the mean total improvement in BI score in both groups exceeded this threshold (24.24 ± 16.61 and 25.67 ± 15.14 in the inpatient and home groups, respectively); this finding also agrees with that of a previous study conducted in Taiwan that reported a mean improvement in BI score of 24.1 in the inpatient PAC program [2]. A Malaysian study reported a BI score improvement of 29.3 in patients with their first-ever acute stroke after 30 days of rehabilitation [33], and another study reported a BI to score improvement of 34.21 at a mean of 58.15 days after beginning the inpatient PAC program [12]. These heterogeneous results may be due to differences in the length of stay in inpatient PAC institutions and patients’ underlying conditions with stroke. As for the efficacy of home-based in the current study, a 6-week post-stroke patients research revealed median total BI improved from 14 to 18 after home-based stroke program [34]; another showed home-based program could significantly shorten the length of hospital stay to 7 days with better daily living score and satisfaction [18]. Home-based occupational therapy in around 30-days post-stroke patients showed better outcomes immediately after discharge [19]. Similar ADL function was noted in home-based and outpatient rehabilitation, with the former being 22 days and the latter being 53 days averagely after stroke [23]. Our study mainly focuses on subacute stroke (2 weeks after stroke in both groups) rehabilitation with a generally equivalent basic condition and comorbidities, as well as comparing therapeutic efficacy by the same executive rehabilitative faculty. Moreover, average improvements in IADL were 1.04 ± 1.29 and 0.88 ± 1.35 in inpatient and home-based groups with no significant difference, and previous inpatient PAC studies showed similar 1 point improvement over IADL score on average [2]. ED5Q presented as patient’s life quality and our study found mean improvements were 1.72 versus 1.64 in inpatient and home-based group, comparing to median 0.66 versus 0.77 in a Denmark study [35]. Significant improvements among all domains of ED5Q during the current inpatient PAC model were acquired with predominantly affecting mobility, self-care and usual activities [12]. Mean improvements in MNA scores, indicating the nutritional status of poststroke patients, were very similar in inpatient and home-based PAC groups in our study (0.70 ± 2.87 versus 0.72 ± 1.28), while the previous study showed average 2-point improvements over MNA in a longer hospitalized PAC program [2]. Besides advanced daily function, life quality and nutritional status, few studies have discussed the categories in daily living activities in detail. One study revealed that scores in the subdomains of mobility, self-care, and usual activities in the EuroQol five dimensions questionnaire were significantly improved at an average of 43.57 days after beginning an inpatient PAC program [36]. Our findings reveal that patients receiving PAC at home had significantly greater improvements in the domains of dressing oneself, self-hygiene, and showering than did those receiving inpatient PAC. This may be because home care has certain advantages offered by a domestic environment, specifically the merging of rehabilitative programs with daily practical circumstances and the greater feasibility of performing and practicing tasks at home, particularly those related to the patient’s use of their bedroom or bathroom.

In our study, the mean length of the PAC program was 27.00 days in the inpatient group and 35.65 days in the home group, which is similar to the reported mean lengths of inpatient rehabilitation of 29.4 days in Thailand, 31.3 days in Ireland, 31.2 days in Switzerland, and 37.1 days in Singapore [28]. The longer duration of the PAC program in the home group may be attributable to patients’ preference for staying at home and being more motivated to participate in rehabilitative programs or the difference in the number of rehabilitative hours per week (15 h/week for the inpatient PAC program vs. only 5 h/week for the home-based PAC model). In our study, the average total number of rehabilitative hours over the entire PAC program was significantly lower in the home-based model than in the inpatient model. The home-based model considerably reduces the need for therapists. Notably, the intensity of the home-based model was only one-third to half of that of the inpatient model, despite a significantly higher number of rehabilitative days, thereby denoting similar or even better functional recovery in patients with stroke.

In our study, the two groups had similar baseline characteristics in terms of sex composition, subtypes of stroke, the severity of the stroke, duration from stroke onset to PAC initiation, ICU care, comorbidities, healthcare utilizations, ADL and nasogastric tube or Foley catheter used at PAC starts. The average duration from stroke onset to PAC initiation was approximately 14 days in our study, which corresponds to previous data: approximately 9.88–17.11 days among different referral hospitals [11] and 15.56 days in another study on patients with stroke aged < 65 years [36]. The inpatient group’s mean age is similar to previous data (approximately 63.01–66 years) [2,36]. A US database study indicated that older individuals tend to receive post-stroke care in skilled nursing facilities [37]. This may also explain why patients were older in the home group in our study. Previously, older patients may have preferred to be institutionalized in the subacute stroke phase; however, older patients with stroke with great rehabilitative potential may have changed their preferences since the home-based PAC program was launched. This speculation must be verified because multiple factors other than age, such as comorbidities and socioeconomic status, can influence patients’ discharge destination after acute stroke treatment. As for patients, who did not complete the PAC program, 15.82% withdrew from the program of their own volition, and 6.21% ceased the program due to disease progression in the inpatient group. Simultaneously, 15.48% withdrew from the program due to self-willingness, and 14.29% discontinued the program due to disease progression in the home-based group. More than two times of patients had quit the PAC program because of disease deterioration in the home-based group than in that of the inpatient group. The mean age of patients who dropped out PAC program under self-preference was older in the home-based than inpatient PAC group (76.08 years vs. 70.43 years). Older patients in home-based stroke rehabilitation were more likely to drop out of the program under self-willingness, while patients may have a higher ratio disrupt the program due to disease progression in the home-based PAC group. However, these are preliminary inferences and require more proof to support them.

Although the home group patients were older in our study, their functional improvements were similar to those of the patients in the inpatient group, with considerably lower total cost. Stroke-related medical expenses amount to approximately USD 375 million [38] annually, and stroke rehabilitation contributes considerably to this cost [13]. Currently, inpatient PAC programs reduce the financial burden by favoring earlier discharge from PAC programs rather than longer and more expensive stays at acute medical institutions [11]. In our study, the mean rehabilitative cost of inpatient PAC programs for each patient with a stroke was NTD 80,975.54 ± NTD 33,213.72 (USD 2699.19 ± USD 1107.12), which was significantly higher than that of the home-based PAC program (NTD 31,617.71 ± NTD 12,557.57 (USD 1053.92 ± USD 418.59)). The inpatient PAC cost in our study is similar to that indicated in a previous study (NTD 81,747–NTD 106,950) involving several referral hospitals [11]. Cost-effectiveness analysis revealed that the home-based PAC model costs NTD 1445.51 ± NTD 1050.53 (USD 48.18 ± USD 35.02) per 1-point increase in BI score, whereas the inpatient model costs NTD 4574.21 ± NTD 4939.84 (USD 152.474 ± USD 164.66) per 1-point increase BI score. In other words, the medical expenses required in the home-based PAC model are considerably lower (approximately one-third lower) than those in the inpatient PAC model to reach the same functional poststroke recovery outcome. We also calculated total medical expense divides IADL/ED5Q/MNA and found significantly lower expense per improved score in the home-based PAC group. The medical delivery system saved approximately 66%, 40% and 56% medical expense per 1-point increase of IADL, ED5Q and MNA in the way of the home-based PAC model. Previous research from Medicare beneficiaries in the USA [39] showed home health care is more cost-effective than PAC in skilled nursing facilities or rehabilitation facilities. However, another systemic review indicated home-based rehabilitation could achieve better health outcomes but is not likely to lead to cost savings [40]. On the other hand, we also calculated the total number of rehabilitative hours divided by the improvement in BI score and found that the rehabilitative duration per BI point improvement was significantly lower (about one-third lower) in the home-based PAC model than in the inpatient PAC model. Little research has discussed the relationship between BI score improvements and the total number of rehabilitative hours. From our findings, we infer that the home-based PAC model encourages patients to execute daily tasks in familiar environments with family participation, which may increase the efficacy and improvement/success rate of patients achieving standards for performing daily activities and self-care. Future studies should further explore this point. Overall, our novel rehabilitative home-based PAC model was observed to save more than 60% in both medical cost and time spent on rehabilitation among patients with stroke.

### Limitations

Our study has several limitations. First, this was a retrospective, single-center study with a small sample size, which may limit the generalizability of our results and some masking preferential factors of patients due to uncollected characteristics. Second, we evaluated only the activities of daily living of patients with stroke. Future studies should include other functional aspects, such as cardiopulmonary capacity, coordination, swallowing function, balance, and hand function and also analyze their cost-effectiveness. Third, we did not include a control group, so the natural course of spontaneous functional recovery in patients with stroke should be considered when interpreting our results. Fourth, age was significantly higher in the home-based PAC group, and a further matched cohort is needed. However, similar daily functional improvement in both groups could still be noted in the original cohort. Future studies can address these limitations.

## 5. Conclusions

The home-based PAC stroke rehabilitation program was non-inferior to the inpatient PAC program in terms of the functional recovery for performing daily activities, life quality and nutritional status. Moreover, patients receiving home-based PAC performed significantly better in the domains of dressing oneself, self-hygiene, and showering. Both rehabilitative cost and the total number of therapeutic hours in the home-based PAC model were significantly lower than those in the inpatient PAC model. The cost-effectiveness and remarkable functional improvements afforded by the home-based program make it a promising rehabilitative model for patients with stroke.

## Figures and Tables

**Table 1:** Comparison of baseline demographic characteristics between the inpatient and home-based post-acute care (PAC) groups.

Variables	Inpatient PAC	Home-Based PAC	*p*-Value
	(*n* = 138)	(*n* = 59)	
Gender (male)	76 (55.10%)	32 (54.24%)	0.914
Age (year-old), median (IOR)	67.5 (57–76)	68.0 (57–78)	0.046 *
Type of stroke			0.195
Ischemic stroke	82.61%	74.58%	
Hemorrhagic stroke	17.39%	25.42%	
Severity of stroke			0.238
mRS 3	28.26%	39.98%	
mRS 4	71.74%	61.02%	
ICU care, n (%)	53 (38.41%)	20 (33.90%)	0.525
Length of day between the onset of stroke and receiving PAC	14.23 ± 7.30	14.07 ± 6.64	0.597
Category of comorbid cardiovascular conditions			
Hypertension	115 (83.33%)	51 (86.44%)	0.583
Acute coronary syndrome	16 (11.59%)	3 (5.08%)	0.156
Diabetes mellitus	51 (36.96%)	26 (44.07%)	0.368
Dyslipidemia	83 (60.14%)	32 (54.24%)	0.441
Atrial fibrillation	19 (13.77%)	10 (16.95%)	0.564
Cerebrovascular disease	34 (24.64%)	12 (20.34%)	0.514
Number of comorbid cardiovascular conditions			0.848
0, n (%)	8 (5.80%)	3 (5.08%)	
1, n (%)	30 (21.74%)	14 (23.73%)	
2, n (%)	42 (30.43%)	18 (30.51%)	
3+, n (%)	58 (42.03%)	24 (40.68%)	
CCI (mean ± SD)	3.38 ± 2.20	3.75 ± 2.19	0.313
0, n (%)	2 (1.45%)	0 (0%)	0.436
1–2, n (%)	51 (36.96%)	19 (32.20%)	
3–4, n (%)	85 (61.69%)	39 (66.10%)	
5+, n (%)	0 (0%)	1 (1.69%)	
Admitted because of stroke in the past 2 years	6 (4.35%)	0 (0%)	0.104
Healthcare utilization in the past 1 year			
Number of outpatient visits	5.03 ± 7.33	7.17 ± 23.12	0.301
Number of hospitalizations	0.18 ± 0.46	0.37 ± 1.26	0.912
Days of hospital stay	1.27 ± 4.98	2.07 ± 6.32	0.908
Number of emergency department visits	0.54 ± 1.02	0.53 ± 1.29	0.186
Initial ADL function at PAC starts			0.157
Totally dependent (BI score 0–20),%	19.57%	11.86%	
Severely dependent (BI score 21–40),%	33.33%	25.42%	
Moderately dependent (BI score 41–60),%	27.54%	30.51%	
Independent,%	19.57%	32.20%	
Nasogastric tube used at PAC starts, n (%)	18 (13.04%)	3 (5.08%)	0.097
Foley catheter used at PAC starts, n (%)	10 (7.25%)	4 (6.78%)	0.907
Length of PAC program, days	27.00 ± 11.44	35.54 ± 10.21	<0.001 ***

ADL, activities of daily life; BI, Barthel index; CCI, Charlson comorbidity index; ICU, intensive care unit; IOR, interquartile range; mRS, modified Rankin scale; PAC, post-acute care; * *p* < 0.05; *** *p* < 0.001

**Table 2:** Functional improvements in the Barthel index score of the inpatient and home-based PAC groups.

Variables	Inpatient PAC (*n* = 138)	Home-Based PAC (*n* = 59)	*p*-Value
Δ Barthel index (BI)	24.24 ± 16.61	25.67 ± 15.14	0.530
Δ Feeding	1.63 ± 2.58	2.03 ± 2.65	0.265
Δ Transfering	4.49 ± 3.64	4.32 ± 3.99	0.717
Δ Ambulation	5.80 ± 5.45	5.51 ± 4.89	0.843
Δ Stair-climbing	3.08 ± 3.33	3.05 ± 3.60	0.953
Δ Dressing oneself	1.27 ± 2.27	2.37 ± 2.84	0.003 **
Δ Bladder control	1.70 ± 3.11	1.44 ± 2.63	0.659
Δ Bowel control	1.16 ± 3.10	0.51 ± 1.52	0.157
Δ Self-hygiene	1.23 ± 2.16	2.12 ± 2.49	0.013 *
Δ Toilet use	3.08 ± 3.04	2.37 ± 2.84	0.133
Δ Showering	0.80 ± 1.93	1.95 ± 2.46	0.001 **

* *p* < 0.05; ** *p* < 0.01; Δ: the difference between the score after PAC completion and that before PAC initiation.

**Table 3:** Comparison of age and improvements of the Barthel index in inpatient and home-based PAC groups.

	Inpatient PAC Group (*n* = 138)				Home-Based PAC Group (*n* = 59)			
Age	<65 Year-Old	65–75 Year-Old	>75 Year-Old	*p*-Value	<65 Year-Old	65–75 Year-Old	<65 Year-Old	*p*-Value
Δ BI < 20	14 (10.14%)	12 (8.70%)	18 (13.04%)	0.177	5 (8.47%)	6 (10.17%)	6 (10.17%)	0.580
Δ BI ≥ 20	34 (24.64%)	22 (15.94%)	19 (13.77%)		16 (27.12%)	10 (16.95%)	10 (16.95%)	

BI, Barthel index; PAC, post-acute care; Δ: the difference between the score after PAC completion and that before PAC initiation.

**Table 4:** Cost-effectiveness, rehabilitative hours and health economics of the inpatient and home-based PAC groups.

Variables	Inpatient PAC (*n* = 138)	Home-Based PAC (*n* = 59)	*p*-Value
Total cost, NTD (USD)	80,975.54 ± 33,213.72 (2699.19 ± 1107.12)	31,617.71 ± 12,557.57 (1053.92 ± 418.59)	<0.001 ***
Cost-effectiveness vs. BI ^∞^ (USD)	4574.21 ± 4939.84 (152.47 ± 164.66)	1445.51 ± 1050.53 (48.18 ± 35.02)	<0.001 ***
Cost-effectiveness vs. IADL ^∞^ (USD)	56,529.97 ± 37,140.36 (1884.33 ± 5.49)	19,426.96 ± 19,888.24 (647.57 ± 662.94)	<0.001 ***
Cost-effectiveness vs. ED5Q ^∞^ (USD)	25,476.70 ± 52,741.59 (849.22 ± 1758.05)	15,623.60 ± 18,564.44 (520.79 ± 618.81)	<0.001 ***
Cost-effectiveness vs. MNA ^∞^ (USD)	35,246.51 ± 61,296.79 (1174.88 ± 2043.23)	15,333.94 ± 24,418.28 (511.13 ± 813.94)	0.001 **
Total rehabilitative hours	57.86 ± 24.52	25.39 ± 7.29	<0.001 ***
Total rehabilitative hours/Δ BI ^†^	3.23 ± 3.38	1.23 ± 0.90	<0.001 ***

NTD: new Taiwan dollar; PAC: post-acute care. ^∞^ Cost-effectiveness was calculated as the total rehabilitative cost divided by improvement in the Barthel index, IADL, ED5Q and MNA scores. ^†^ Total rehabilitative hours/Δ BI was calculated as the total number of rehabilitative hours divided by improvement in the Barthel index score. ** *p* < 0.01; *** *p* < 0.001.

## Data Availability

Data are available on reasonable request by email to Dr. Willy Chou (corresponding author).
